# Prevalence and associated risk factors of *Strongyloides stercoralis* infection in Lower Myanmar

**DOI:** 10.1186/s41182-018-0126-5

**Published:** 2018-12-18

**Authors:** Myo Pa Pa Thet Hnin Htwe Aung, Akina Hino, Khine Mar Oo, Kyu Kyu Win, Haruhiko Maruyama, Wah Win Htike, Eiji Nagayasu

**Affiliations:** 1 Department of Microbiology, University of Medicine 1, No. 245 Myoma Kyaung Street, Lanmadaw Township, Yangon, Myanmar; 2 Division of Parasitology, Department of Infectious Diseases, Faculty of Medicine, University of Miyazaki, 5200 Kihara, Kiyotake, Miyazaki, 889-1692 Japan

**Keywords:** *Strongyloides stercoralis*, Prevalence, Myanmar, Agar plate culture, Molecular assays

## Abstract

**Background:**

Strongyloidiasis is prevalent in Southeast Asian regions along with other soil-transmitted helminthiases, but only limited present-day data was available for Myanmar.

**Methods:**

A prevalence survey for *Strongyloides stercoralis* infection was conducted among villagers in rural areas of three townships located in the Lower Myanmar during 2014–2016 by agar plate culture method in combination with specific identification by molecular assays. Risk factors associated with *S. stercoralis* infection were assessed by analyzing questionnaires obtained from study participants.

**Results:**

*Strongyloides stercoralis* was identified in 40 out of 703 participants (5.7% overall prevalence). The highest prevalence (14.4%) was observed in Htantabin, while other two communities (Thabaung and Thanlyin) had much lower prevalence (2.2 and 2.5%, respectively). Infection was relatively rare (1.2%) in younger generations under 20 years compared to older generations (9.5%). Even in Htantabin, none of the female residents under age 40 (*n* = 33) had infection. In adult Htantabin residents, those who answered that they do not wear shoes regularly had an elevated risk of infection (odds ratio = 2.50, 95% confidence interval = 1.03–6.08).

**Conclusions:**

This study showed that there is still an on-going transmission of strongyloidiasis in Lower Myanmar. It is highly desirable that the soil should be free of fecal contamination by improving the management of fecal waste. Meanwhile, health education to promote shoe-wearing would be beneficial to reduce the risk of transmission, especially for those who have frequent and intense contact with soil.

## Background

*Strongyloides stercoralis* has been described as one of the most neglected soil-transmitted helminths (STHs) among the neglected tropical diseases (NTDs) [1]. Although infection is acquired via contaminated soil, similar to major STHs, namely *Ascaris lumbricoides*, *Necator americanus*, *Ancylostoma duodenale*, and *Trichuris trichiura*, epidemiological information on *S. stercoralis* infection is scarce [2]. The primary route of infection of *S. stercorailis* is transdermal (infective larvae penetrate the skin), similar to hookworms (*N. americanus*, *A. duodenale*), but differing from *A. lumbricoides* and *T. trichiura*, which are transmitted orally by ingestion of infective eggs.

Strongyloidiasis has a cosmopolitan distribution. Recently, it was estimated that at least 370 million are infected worldwide [3]. It affects 10–40% of the population in many tropical and subtropical countries [4]. In resource-poor countries with ecological and socioeconomic settings conducive to the spread of *S. stercoralis*, infection rates can be high, up to 60% [4].

Human strongyloidiasis is caused by two nematode species that belong to the genus *Strongyloides*: *S. stercoralis* is the most common and globally distributed human pathogen of clinical importance [5], while *S. fuelleborni* is found sporadically in Africa (*S. fuelleborni fuelleborni*) and Papua New Guinea (*S. fuelleborni kellyi*) [6].

Although many cases of strongyloidiasis are asymptomatic, infection may be severe and even life-threatening in immunocompromised individuals. The unique ability of this nematode to replicate in the human host permits the cycles of autoinfection, leading to chronic disease that can last for several decades [6]. For treatment, ivermectin is more effective than thiabendazole or albendazole and considered the first-line therapy [7]. Myanmar currently has government-run mass drug administration (MAD) programs in place for both STH and lymphatic filariasis [8]. The former uses albendazole and the latter uses albendazole and diethylcarbamazine citrate [8]. Ivermectin, the first-line drug to treat strongyloidiasis, has not been used for the purpose of MDA.

Strongyloidiasis is frequently underdiagnosed, since many infections remain asymptomatic and conventional diagnostic tests based on parasitological examination are not sufficiently sensitive. The difficulty in diagnosing *S. stercoralis* infection is one of the reasons why up-to-date, accurate information on its geographic distribution in endemic regions and the total global burden is lacking. In Myanmar, there are few up-to-date data on the prevalence of *Strongyloides* and most of the previous studies were done by low-sensitive routine microscopic examinations.

During 2014–2016, we conducted an epidemiological survey to determine the prevalence of strongyloidiasis among villagers in rural areas of three divisions in Lower Myanmar. We used agar plate culture method in combination with specific identification of the isolated nematode by molecular assays. As a part of this project, molecular phylogenetic analyses of *S. stercoralis* worms isolated from these villagers were conducted [9]. In this paper, we described an epidemiological situation of strongyloidiasis in Lower Myanmar that was not included in our previous publication [9]. The results of this study will contribute to the development of appropriate strategies in the control of STH.

## Methods

### Study design

**Fig. 1:**
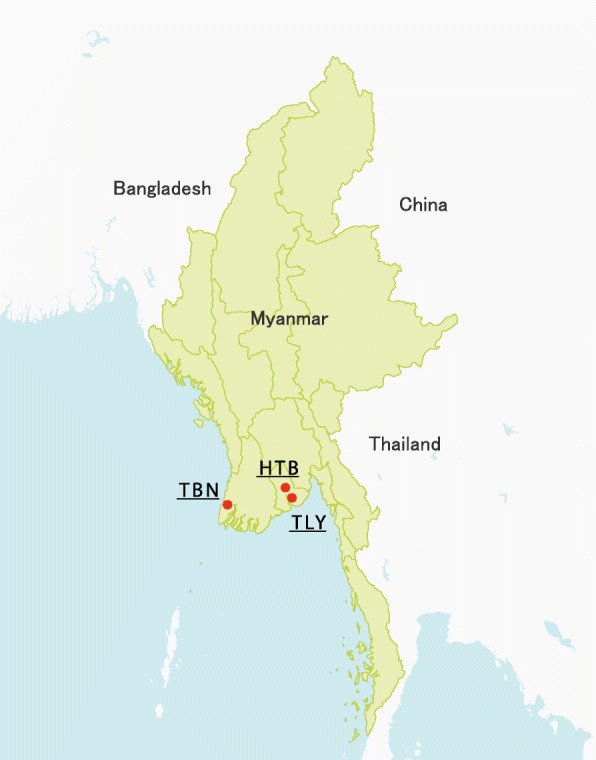
Stool sampling locations in Lower Myanmar. HTB: Htantabin, TBN: Thabaung, and TLY: Thanlyin

Cross-sectional descriptive study was carried out from January 2014 to January 2016. The study areas consisted of villages of Htantabin Township (Yangon Division), Thanlyin Township (Yangon Division), and Thabaung Township (Ayeyarwady Division). The locations of these sampling sites are shown in Fig. 1.

Htantabin Township is located on the western border of central Yangon Region, Myanmar. There are 59 village tracts in the township consisting of 230 villages. Overall population is 120,454 (2008). Thabaung is a town in the Pathein District, the Ayeyarwady Division of south-west Myanmar. There are 70 village tracts in the township consisting of 421 villages. Total population is 154,400 (2014). Thanlyin is a major port city of Myanmar, located across Bago River from the city of Yangon. Thanlyin Township comprises 17 quarters and 28 village tracts. The total population is 181,000 (2009).

A multistage sampling method was used as the sampling procedure. Villages from each township were selected by the lottery method of randomization. Approximately 250 villagers per township were selected. Villagers who received any anti-helminthic treatment within 3 months prior to the study and villagers presenting with diarrhea at the time of sampling were excluded from the study.

### Collection of fecal specimens

Plastic containers (clean, wide mouth screw-capped containers) and plastic bag were provided to those who agreed to participate. The participants were interviewed using structured questionnaires to obtain information such as their age, gender, education status, occupation, monthly income, regular wearing of shoes, and close contact with dogs (potential reservoir host). Overall, 703 villagers from three townships who provided both stool and completed a set of questionnaires were included in this study.

Stool specimens were transported using an air-conditioned car with inside temperature of about 25 °C. Transportation took about 2, 2, and 5 h from each sampling site (Htantabin, Thanlyin, and Thabaung, respectively) to the laboratory.

### Fecal examinations

The agar culture method for detection of *S. stercoralis* and worm lysate preparation procedures were based on previous publications [9, 10]. Agar plates were cultured at room temperature (approximately 25 °C) for 4 days. Then, they were checked daily under a stereomicroscope to detect any nematode. Larvae or adult stages of nematodes found on the agar plates were picked up by a worm picker (fine needle made from tungsten) into polymerase chain reaction (PCR) tubes containing 10 μl of worm lysis solution that was prepared by mixing 0.5 volume of proteinase K (> 600 mAU/ml solution, catalog no. 19131, Qiagen), 0.5 volume of 1 M dithiothreitol (DTT), and 10 volumes of DirectPCR Lysis Reagent (Tail) (catalog no. 101-T, Viagen Biotech Inc.). The nematodes were lysed by incubation at 60 °C for 20 min. They were then incubated at 95 °C for 20 min to inactivate proteinase K. The tubes containing worm lysate were stored at − 30 °C until used.

Presence of STHs other than *S. stercoralis* was assessed by direct wet mount microscopy.

### PCR and DNA sequencing

A portion (935 bp) of 18S rDNA gene which corresponds to positions − 2–933 of AF279916 (GenBank) was amplified using a primer pair 988F (5′-ctcaaagattaagccatgc-3′) and 1912R (5′-tttacggtcagaactaggg-3′) [11]. The same primer, 1912R, was used for the sequencing reaction. We used a 333-bp region which spanned between positions 315–683 for identification of species as *S. stercoralis* [9].

### Statistical analysis

After collection of data, data cleansing, data compilation, data processing, and data analysis were done by using statistical Excel and SPSS version 18.0.

## Results

### Study population

In total, 703 individuals from three townships were included in this study. The characteristics of the study population are summarized in Table 1. Among them, 342 (48.6%) were male and 358 (51.4%) were female. The median age of all participants was 28 years with a range of 1–105. In Htantabin, 31.4% of the adult (equal or more than 20 years old) participants were farmers. On the other hand, none of the adult participants were farmers in Thabaung and Thanlyin.

**Table 1:** Characteristics of the study populations

	Htantabin (HTB)	Thabaung (TBN)	Thanlyin (TLY)
	*n* = 195	*n* = 271	*n* = 237
Median age (range)	45 (2–84)	13 (2–68)	13 (1–105)
Gender (male/female)	102/93	139/132	101/136
Median monthly income (kyats)	150,000	196,000	100,000
Proportion (%) of farmer among adult participants	31.4	0	0

### Strongyloidiasis prevalence determined by an agar plate culture method

**Fig. 2:**
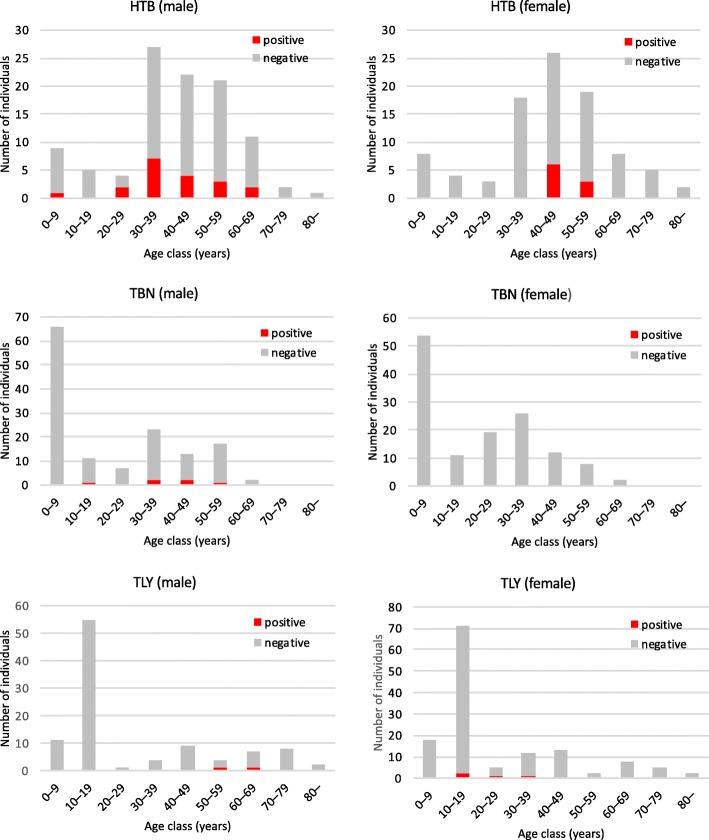
Number of *S. stercoralis* positive and negative individuals by age group

Nematodes were identified from 6.3% (44 out of 703) of the fecal samples by an agar plate culture method. Up to eight nematodes per sample were collected and subjected to sequencing analysis. The presence of *S. stercoralis* were confirmed in 40 out of 703 fecal samples, based on the partial 18S rDNA sequences. Therefore, the overall prevalence of strongyloidiasis among the study population was estimated to be 5.7%. The highest prevalence was seen in Htantabin (28 out of 195, 14.4%), followed by Thanlyin (6 out of 237, 2.5%) and Thabaung (6 out of 271, 2.2%). Prevalence by gender and age group is presented in Fig. 2. In Thabaung, none of the 132 female participants were infected. In Htantabin, no female participants below the age of 40 were infected.

### Risk factors for *S. stercoralis* infection

A risk factor analysis was conducted only for Htantabin residents because of the low strongyloidiasis-positive case numbers, identified in the present study, in the other two communities. Being 20 years or more had the highest odds ratio (4.75) for infection, although the difference was considered not statistically significant (*p* = 0.135). Other variables were tested only for adult participants, because there was only one positive case among participants below the age of 20, and some variables such as monthly income were relevant only in adult population. Among the variables tested, only “not-wearing shoes regularly” was associated with *S. stercoralis*-positive test results with statistical significance (Table 2).

**Table 2:** Correlation of *S. stercoralis* infection and sociodemographic and other characteristics

Variable	No. Infected	Odds ratio [95% CI]	*p*
Age†			
< 20 years	1/26 (3.8%)		
≥ 20 years	27/169 (16.0%)	4.75 [0.62–36.58]	0.135
Gender			
Female	9/72 (12.5%)		
Male	18/70 (25.7%)	2.06 [0.87–4.89]	0.14
Occupation			
Other than farmer	15/116 (12.9%)		
Farmer	12/53(22.6%)	1.97 [0.85–4.57]	0.119
Monthly income			
≥ 150 K kyats	14/94 (14.9%)		
< 150 K kyats	13/75 (17.3%)	1.20 [0.52–2.73]	0.679
Education			
Less than middle school	23/128 (18.0%)		
Middle school or more	4/41 (9.8%)	0.49 [0.16–1.52]	0.326
Regular wearing of shoes			
Yes	17/115 (14.8%)		
No	10/37 (27.0%)	2.50 [1.03–6.08]	0.045*
Close contact with dogs			
No	24/115 (20.9%)		
Yes	3/30 (10.0%)	0.53 [0.15–1.90]	0.428
Presence of other STHs			
No	24/148 (16.2%)		
Yes	3/21 (14.3%)	0.86 [0.24–3.15]	1.000

†Data from all age groups, otherwise data from only adults (20 years old or more) were used

*p < .05

*No.* number, *CI* confidence interval, *STH* soil-transmitted helminths

## Discussion

Although chronic strongyloidiasis is often asymptomatic or accompanied only by low-grade gastrointestinal symptoms, it can also be fatal in immunocompromised or corticosteroid-treated individuals [12]. Awareness of strongyloidiasis is important, especially to prevent hyperinfection and disseminated strongyloidiasis. In Myanmar, however, the presence of *S. stercoralis* has not been well recognized. Direct wet mount microscopy and Kato-Katz technique are commonly used in prevalence studies of geohelminthes, but they are inadequate for *S. stercoralis* detection*.*

In this study, we used the agar plate culture, that is one of the most sensitive parasitological methods to detect *S. stercoralis* infection [13, 14], and the overall prevalence of strongyloidiasis among our study population in Lower Myanmar was found to be 5.7%. It was higher than the prevalence reported from previous studies, such as 3.8% of children from Yangon Children Hospital in 1987 [15], 0.8% of patients attending No. 2 Military Hospital during 2000–2001 [16], and 1.67% of children from North Okkalapa Township, Yangon, in 2004 [17]. However, our prevalence was lower than the values reported from some older studies, such as 15 and 13% among miners working at Mawlamyaing Antimony Mine and No. 1 copper project at Monywa, respectively, in 1981 [18].

The overall prevalence of 5.7% is rather low compared to recent prevalence reported from other Southeast Asian countries, such as Lao PDR (41.0%) [19] and Cambodia (21.0%) [20]. This may be due to the relatively large proportion of non-farmers included in our study.

Among the three townships, Htantabin had the highest prevalence (14.4%). Even there, none of the female participants under the age of 40 were infected (0 out of 33). This was a great contrast to the male participants belonging to the same age groups (10 out of 45; 22.2%). This may indicate relatively low chance of transmission at home considering that males and females share the same domestic environment. We hypothesize that the higher prevalence in males might be due to more chance for them to acquire infection during outdoor activities such as farming compared to females who tend to spend more time to carry out their works at home.

Persons who do not wear shoes regularly had higher risk of infection. The association of failure to wear shoes and *S. stercoralis* infection has been reported in other studies such as those conducted in Peruvian Amazon [21] and Lao PDR [22]. There is an urgent need to improve environmental sanitation (reducing fecal contamination in soil) to prevent soil-to-skin transmission of *S. stercoralis*. More investigation is needed to elucidate the degree and mechanisms of fecal contamination of the soil in these areas. Meanwhile, education to emphasize the wearing of shoes should be given especially to those who have frequent and intense contact with soil, such as farmers.

We would like to conduct similar studies in other areas of Myanmar, and we hope that such studies will be effectively used to develop the appropriate strategies for the control of STH including strongyloidiasis.

## Conclusions

*S. stercoralis* infection is still prevalent among villagers in some rural areas of the Lower Myanmar. Healthcare professionals need to be aware of potential *S. stercoralis* infection among the residents living in these endemic areas, especially when treating them with corticosteroids for a variety of medical reasons, to avoid the risk of life-threatening hyper-infection. Health education and motivation for good hygienic practices, improved sewage disposal techniques, and the habit of wearing of shoes would also be salient factors to reduce the risk of infection.

## Abbreviations

DTT: Dithiothreitol
NTDs: Neglected tropical diseases
PCR: Polymerase chain reaction
rDNA: Ribosomal deoxyribonucleic acid
STHs: Soil-transmitted helminths
